# The Institutional Black Box of Friction Between Digital Agility Demands and Clinical Bureaucracy in Hospital Social Media Management

**DOI:** 10.12688/f1000research.180694.1

**Published:** 2026-05-27

**Authors:** Renaldi Renaldi, Sri Ulfa, Rita Rosa, Erlin Eka Wulandari, Sulistiani Sulistiani, Muhammad Rosyid Ridho, Rani Surya Resiana

**Affiliations:** 1 Health Policy and Management, Gadjah Mada University, Yogyakarta, Special Region of Yogyakarta, Indonesia; 2 Economic and Business, Gadjah Mada University, Yogyakarta, Special Region of Yogyakarta, Indonesia; 3 Islamic Economy and Halal Industry The Graduate School, Gadjah Mada University, Yogyakarta, Special Region of Yogyakarta, Indonesia

**Keywords:** Digital Agility, Institutional Logics, Dark Social, Qualitative Content Analysis, Hospital Management, Social Media Marketing, Electronic Word-of-Mouth.

## Abstract

**Background:**

In the contemporary healthcare landscape, hospitals face a paradox between the need for digitally agile, emotionally engaging communication and the rigid, risk-averse nature of clinical bureaucracy. While social media enables narrative-based engagement, its implementation in hospitals remains constrained. This study aims to examine how institutional dynamics shape the implementation of narrative content and to explain the gap between audience engagement and observable electronic word-of-mouth (e-WOM).

**Methods:**

This study employs a qualitative approach using Directed Qualitative Content Analysis (DQCA). Data were collected through in-depth stimulated recall interviews with hospital audiences and internal management, and analyzed using ATLAS.ti to identify thematic patterns related to institutional constraints, content implementation, and audience responses.

**Findings:**

The findings reveal two key mechanisms. First, narrative content is constrained by institutional logics and bureaucratic processes, particularly approval bottlenecks, resulting in delayed, modified, or diluted implementation. Second, although narrative content generates high affective audience engagement, this does not consistently translate into public e-WOM. This engagement–behavior gap is explained by the role of dark social, where content is shared through private channels due to privacy concerns, social norms, and perceived risks.

**Conclusions:**

This study contributes by proposing a conceptual framework of institutional friction and introducing a practical Quality Control (QC) checklist to support the implementation of narrative content in hospital settings. The findings highlight the need to align communication strategies with institutional realities while recognizing that conventional metrics may underestimate the true impact of digital engagement due to the prevalence of dark social.

## 1. Introduction

In the contemporary healthcare landscape, hospitals face a profound paradox: the growing demand for digital agility and emotionally engaging communication increasingly conflicts with the rigid, risk-averse nature of clinical bureaucracy. While digital platforms require fast, adaptive, and audience-oriented content strategies, hospital communication practices remain shaped by institutional logics that prioritize accuracy, compliance, and reputational control. This tension creates challenges not only in how communication is produced, but also in how it is experienced and responded to by audiences.

This paradox became more visible during the COVID-19 pandemic, which disrupted healthcare systems globally, not only in clinical delivery but also in service access and utilization. A report by the World Health Organization indicates that approximately half of essential health services experienced disruptions at the peak of the pandemic ( WHO, 2021). In Indonesia, this disruption was reflected in a significant decline in healthcare utilization, particularly outpatient services, which dropped by 30–50% in the early phase of the pandemic. These conditions placed hospitals under dual pressure: operational strain and the strategic need to restore patient trust and engagement in an increasingly digital environment.

This phenomenon is also evident in large hospitals in Indonesia, including a private hospital in Yogyakarta. Data shows that outpatient visits declined from 311,198 in 2019 to 200,284 in 2021 (−35.6%), while inpatient services dropped by 46% from 21,857 to 11,793 patients during the same period ( Dinas Kesehatan Kota Yogyakarta, 2024). Although recovery trends are observable, by the end of 2023 service volumes (270,508 outpatient visits and 16,466 inpatient cases) had not fully returned to pre-pandemic levels. This persistent recovery gap suggests that restoring healthcare utilization cannot rely solely on clinical improvements, but also requires more effective communication strategies that can rebuild trust and influence patient behavior.

In this context, *trust*, especially in the digital environment, is a key factor. Studies show that *digital trust* in healthcare is not only influenced by the quality of information, but also by the way it is communicated ( Beldad & Hegner, 2018; Zhao *et al*., 2020). Thus, the effectiveness of communication is no longer purely informative, but must also be able to build the psychological engagement of the audience.

In line with that, social media marketing ( *SMM*) has developed into a strategic instrument in hospital management. Digital marketing has been proven not only to convey information, but also to play a role in building public relationships and trust ( Ayuningtyas, 2022). A number of studies show that social media activities correlate with increased patient visits and the formation of *brand equity* and *trust* ( Udayana *et al*., 2024; Dzakiyya & Hijrah Hati, 2024; Kurniawan & Berlianto, 2022).

Among the various platforms, Instagram is becoming a relevant medium because it is visual and narrative-based. This platform allows the delivery of messages that are not only informative but also persuasive through *storytelling.* However, the effectiveness of the resulting communication is not always in line with the intensity of content production.

As shown in Table 1, there is a noticeable difference in content performance. Informative-rational content dominates in numbers, but results in relatively low engagement rates. In contrast, the less frequently produced narrative-emotional content indicates high engagement and viral potential. These findings indicate a gap between content strategy and audience response, and suggest that content management is not yet fully evidence-based.

**Table 1.** Comparative data on the allocation and performance of social media content at the investigated hospital.

Data Indicator	Narrative Content	Documentation Content
Representattive Sample	Retirement Farewell Video (dr. Edhi Dharma)	Workshop & Official Visit Video
Focus of Material	Patient-Centric (Humanistic/Emotional)	Company-Centric (Ceremonial/Reporting)
Production Proportion (Data Basis: Phase 1 Audit, Aug–Nov 2025)	14% (13 of 91 Posts)	86% (78 of 91 Posts)
Average Shares (e-WOM) (Data Basis: Phase 2 Audit, June 2024–July 2025)	> 400 (Up to 2,568 Shares)	0–1

**Source:** Author’s own creation

Theoretically, emotions are recognized as an important factor in influencing the audience’s response. Emotional *engagement* acts as a mediator between content and sharing behavior ( *electronic word-of-mouth*/e-WOM). However, most of the research was conducted on non-health sectors such as retail and e-commerce, so the generalization of the *high-trust hospital sector* was limited. In addition, previous studies have tended to emphasize cognitive mediators such as *trust*, while affective mechanisms are still underexplored in the context of healthcare.

Nevertheless, the gap between content performance and the strategy implemented cannot be explained solely from the perspective of content characteristics or audience responses. In the context of hospital organizations, the production and distribution of social media content is also influenced by the internal dynamics of the institution, including bureaucratic structures, operational standards, as well as institutional logic that emphasizes accuracy and compliance with regulations ( Thornton *et al*., 2012). These characteristics, while important in maintaining the quality of medical services, have the potential to limit flexibility in adopting a more narrative and emotional approach to communication. As a result, the implementation of content strategies often faces structural barriers that lead to the dominance of rigid and unengaging content.

Furthermore, the high level of audience engagement with certain content is not always directly proportional to the level of *electronic word-of-mouth* (e-WOM) that is publicly observed. This phenomenon can be explained through the concept *of dark social*, which is the activity of sharing content that occurs through private channels such as instant messaging or messaging applications, which cannot be tracked through public metrics of social media ( Madrigal, 2012; RadiumOne, 2016). In the context of health services, where privacy issues and information sensitivity are very high, the possibility of *dark social sharing is greater. This creates a dissonance between high engagement and low visibility of e-WOM, thus obscuring the evaluation of the effectiveness of hospital digital communication strategies.*

Based on this description, this study not only seeks to understand the effectiveness of content in terms of audience response, but also examines how institutional dynamics and structural limitations affect the implementation of digital communication strategies, as well as how audience sharing behavior may not be fully observed through conventional indicators.

Specifically, this study is guided by two primary research questions:(1) How do institutional logics and bureaucratic bottlenecks hinder the routine implementation of narrative content in hospitals? (2) Why does high affective audience engagement fail to translate into public e-WOM, and what is the role of *dark social in this dissonance?*

## 2. Literature review

### 2.1 Theoretical review

This research is grounded in a theoretical perspective that emphasizes the role of affective and narrative processes in digital marketing communication, particularly within institutional contexts. Rather than relying on cognitive-processing frameworks that are typically associated with quantitative approaches, this study adopts a qualitative lens to understand how meaning, emotion, and institutional structures shape digital content and audience responses.

Central to this study is the concept of narrative transportation, which explains how individuals become cognitively and emotionally immersed in story-based content. When audiences are “transported” into a narrative, they are more likely to experience emotional engagement and internalize the message, which in turn influences their attitudes and behavioral tendencies, including content sharing. This framework is particularly relevant in social media environments such as Instagram, where visual storytelling enables the integration of emotional and experiential elements in communication ( Green & Brock, 2000; Escalas, 2004).

In digital marketing contexts, audience responses are not merely the result of content exposure, but are mediated by internal processes such as emotional engagement. Prior studies have shown that emotional engagement plays a crucial role in shaping interactive behaviors, including liking, commenting, and sharing, which are central indicators of social media performance ( Dessart *et al*., 2015; Hollebeek *et al*., 2014). From this perspective, storytelling is not only a communication technique but also a mechanism that activates emotional resonance and drives engagement outcomes.

However, the production of digital content especially in organizational settings is not solely determined by communicative effectiveness. It is also shaped by broader institutional structures and norms. Therefore, this study incorporates the perspective of institutional logics, which highlights how organizational behavior is influenced by competing belief systems, rules, and values embedded within institutions. In the context of hospitals, professional, bureaucratic, and managerial logics often coexist, shaping how communication content is produced, approved, and disseminated ( Thornton *et al*., 2012; Friedland & Alford, 1991).

This perspective is particularly important in explaining why emotionally engaging, narrative-driven content may be underproduced despite its effectiveness. Institutional constraints such as formal procedures, risk aversion, and reputational considerations can limit the adoption of more affective and patient-centered storytelling approaches. As a result, there is often a gap between what is effective in engaging audiences and what is actually produced by organizations.

Although prior research in healthcare communication has primarily focused on cognitive dimensions such as trust, this study argues that affective and institutional dimensions must be considered simultaneously. Trust and emotion are not mutually exclusive; rather, they interact in shaping how audiences interpret and respond to health-related content ( Beldad & Hegner, 2018; Zhao *et al*., 2020). By integrating narrative transportation and institutional logics, this research provides a more comprehensive framework to understand the dynamics of digital communication in high-trust sectors such as healthcare.

### 2.2 Social Media marketing in healthcare

Social media *marketing (SMM*) has developed into a strategic instrument in hospital management. Not only does SMM function as a means of conveying information, SMM also plays a role in building public trust in health institutions ( Ayuningtyas, 2022). In the post-pandemic context, this role has become increasingly important as people’s dependence on digital information increases.

A number of studies show that social media activities have a relationship with increasing patient visits and building *brand equity* and *trust* ( Udayana *et al*., 2024; Dzakiyya & Hijrah Hati, 2024; Kurniawan & Berlianto, 2022). Nevertheless, most of the studies still focus on the direct relationship between social media activity and outcomes, without explaining the underlying psychological mechanisms of the relationship.

### 2.3 Narrative content and storytelling in social media

Narrative content or *storytelling* is a communication approach that conveys messages through storylines that involve emotional aspects. Compared to informative content, narrative content is more effective in grabbing attention and building audience engagement.

In practice, the use of *storytelling* on hospital social media is still limited, although there are indications that this type of content has higher performance than informative content. This shows the potential that has not been utilized optimally, while at the same time emphasizing the need for a deeper understanding of the working mechanisms of narrative content in the context of health services.

### 2.4 Emotional engagement

Emotional engagement refers to an individual’s affective response to a piece of content that affects the level of attention, interaction, and connection to the message. In the context of social media, emotions play an important role in driving user engagement.

Research shows that emotional engagement can increase an individual’s likelihood of interacting and sharing content. Nevertheless, most studies still place this variable as a consequence, rather than as a mediator that explains the relationship between content characteristics and audience behavior. In addition, in the context of health services, research emphasizes more on the aspect of trust, so the role of emotional engagement is still less of a concern.

### 2.5 Electronic word-of-mouth (e-WOM)

*Electronic word-of-mouth* (e-WOM) is one of the important indicators of the effectiveness of digital communication. e-WOM reflects the active involvement of the audience in disseminating information to other users, which in the context of healthcare has significant implications for the perception and decision to use the service. A number of studies show that social media activities contribute to the formation of *trust* and intention to visit patients ( Udayana *et al*., 2024; Dzakiyya & Hijrah Hati, 2024; Kurniawan & Berlianto, 2022). Nevertheless, the relationship between content characteristics and e-WOM is often explained directly, without examining the underlying psychological mechanisms.

### 2.6 Institutional logics and bureaucratic constraints in healthcare communication

Healthcare organizations operate within complex institutional environments characterized by multiple, often competing, logics. According to Friedland and Alford (1991), institutional logics shape organizational behavior by embedding norms, values, and rules that guide decision-making processes. In the context of hospitals, professional logics (e.g., medical ethics, risk aversion) frequently coexist with managerial and regulatory logics, creating tensions that influence communication practices.

These institutional dynamics often manifest as bureaucratic constraints, including hierarchical approval procedures, strict compliance requirements, and limited organizational flexibility ( Scott, 2014; Reay & Hinings, 2009). In digital communication, particularly social media, such constraints can hinder the timely and creative implementation of narrative content. Hospitals tend to prioritize accuracy, risk mitigation, and reputational protection, which may result in delayed, diluted, or overly formalized messaging.

Empirical studies suggest that healthcare organizations are generally more conservative in adopting innovative communication strategies compared to other sectors, due to heightened sensitivity to public scrutiny and legal implications ( Kreps & Neuhauser, 2010). As a result, narrative-based content despite its potential effectiveness may not be routinely implemented or may be significantly altered during the approval process.

This indicates that institutional logics and bureaucratic bottlenecks play a critical role in shaping not only whether narrative content is used, but also how it is ultimately presented to audiences.

### 2.7 Dark Social and private information sharing

While the engagement–behavior gap explains the absence of observable public actions, it does not necessarily imply that no sharing occurs. Instead, a growing body of research highlights the role of Dark Social, a term popularized by Madrigal (2012), referring to the sharing of content through private digital channels such as messaging applications, email, and direct messages.

Dark Social represents a form of information diffusion that is largely invisible to traditional analytics tools, yet highly influential in shaping perceptions and decision-making. In contrast to public platforms, private channels offer greater control over audience, reduced social risk, and enhanced privacy, making them particularly suitable for sharing sensitive or personal information.

In healthcare contexts, where topics may involve illness, vulnerability, or stigma, individuals may prefer to share information within trusted networks rather than in public spaces. This aligns with findings in digital communication research suggesting that users strategically choose communication channels based on perceived risk and relational context ( Bansal *et al*., 2010; Berger, 2014).

Thus, Dark Social provides a critical explanation for the observed dissonance between high emotional engagement and low levels of public e-WOM. Rather than indicating disengagement, the absence of public sharing may reflect a shift toward private, less visible forms of communication.

Integrating the concept of Dark Social into the framework allows for a more comprehensive understanding of how digital health communication operates beyond publicly observable metrics.

### 2.8 Theoretical framework

**Figure 1.**
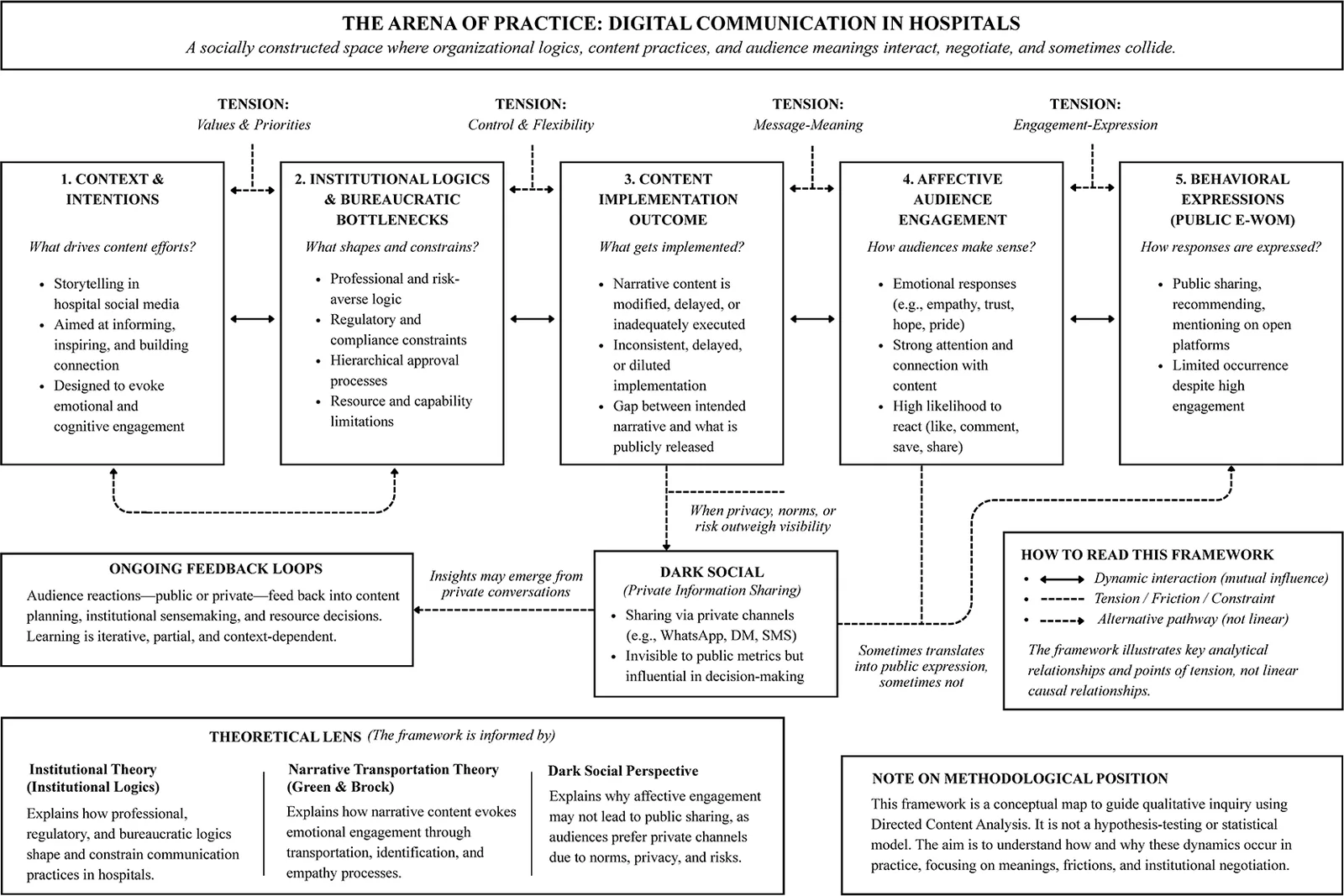
The conceptual framework of institutional friction.

Figure 1 presents a conceptual framework of institutional friction, developed to guide the qualitative analysis using a directed content analysis approach. Rather than representing a statistical or causal model, the framework serves as a process-oriented analytical map that illustrates how narrative content practices interact with institutional constraints and audience dynamics within the hospital context.

The arrows in the framework do not indicate deterministic causal relationships, but instead reflect analytical flows and points of tension between key constructs, including narrative intentions, institutional logics, implementation outcomes, affective engagement, and behavioral responses. In particular, the framework highlights the frictions between affective audience engagement and observable public e-WOM, emphasizing the role of privacy concerns, social norms, and perceived risks.

Furthermore, the inclusion of dark social captures the possibility that audience responses may shift into private communication channels, suggesting that engagement does not always translate into visible metrics. Thus, the framework is intended to map interpretive relationships and thematic patterns, rather than to test hypotheses or quantify causal effects.

## 3. Research methodology

### 3.1 Research design

This study adopts a qualitative research design grounded in an interpretivist paradigm ( Denzin, 1978; Guba & Lincoln, 1994; Patton, 2015; Saunders *et al*., 2019), which assumes that reality is socially constructed and context-dependent. This philosophical stance is particularly appropriate for examining complex, experience-based phenomena such as narrative content adoption in digital healthcare communication. The qualitative approach enables the researcher to capture rich, in-depth insights into *how* and *why* narrative transportation influences audience engagement, emotional responses, and meaning-making processes within the social media ecosystem. Unlike quantitative approaches that prioritize measurement and generalization, this design allows for the exploration of subjective interpretations, institutional dynamics, and contextual nuances that shape communication effectiveness in hospital settings.

Furthermore, the study is situated within the context of hospital management in Indonesia as a developing country, where digital transformation in healthcare communication is still evolving. By integrating both audience and organizational perspectives, this research provides a holistic understanding of the interaction between content production strategies and audience reception.

### 3.2 Informant selection and triangulation strategy

This study employs purposive sampling, combining criterion sampling and maximum variation sampling to ensure both relevance and diversity of perspectives. The inclusion criteria required informants to have direct exposure to and engagement with hospital social media content, particularly those who actively interact with the hospital’s official Instagram account.

Maximum variation sampling was applied to capture heterogeneity across demographic and professional backgrounds, enabling a more nuanced understanding of audience responses across generational and occupational segments. A total of ten informants participated in the study, consisting of eight audience members and two internal management representatives ( Hennink *et al*., 2017; Hennink & Kaiser, 2022). This composition was intentionally designed to facilitate data triangulation, integrating: *Outside-in perspectives* (audience experiences and expectations), and *Inside-out perspectives* (organizational strategies and constraints). This dual perspective is critical for identifying gaps between narrative intention and audience reception, as well as uncovering institutional barriers in digital health communication. The detailed characteristics of the research informants are presented in Table 2.

**Table 2.** Characteristics of research informants.

Informant Pseudonym	Gender	Age	Background	Segment
R-001	Female	28 years	Health Student	Audience (Millennial)
R-002	Female	30 years	Private Sector Employee	Audience (Millennial)
R-003	Female	26 years	Health Student	Audience (Millennial)
R-004	Male	22 years	Content Creator	Audience (Gen Z)
R-005	Female	26 years	Community Health Center Staff	Audience (Millennial)
R-006	Male	26 years	Private Social Media Team	Audience (Millennial)
R-007	Female	23 years	Nursing Student	Audience (Gen Z)
R-008	Male	22 years	Business Communication Student	Audience (Gen Z)
R-009	Female	26 years	Content Team	Internal Management
R-010	Female	48 years	Head of Public Relations	Strategic Decision Maker

**Source:** Author’s own creation

### 3.3 Data collection instrument

Data collection was conducted between January and March 2026 using in-depth Stimulated Recall Interviews (SRI) ( Griffioen *et al*., 2020; Lyle, 2003). This technique is particularly effective in uncovering tacit knowledge and capturing participants’ real-time cognitive and emotional responses, especially in media consumption contexts where reactions are often subconscious and difficult to articulate retrospectively.

The interview protocol was deliberately designed to include a maximum of five open-ended core questions. This structure ensured depth of inquiry while minimizing respondent fatigue and maintaining focus on the research objectives. Each interview session incorporated visual and narrative stimuli to enhance recall accuracy and ecological validity. Informants were exposed to two contrasting types of content from the hospital’s official Instagram account:

1) A high-performing narrative-driven video, portraying a clinician’s post-retirement story, designed to evoke emotional engagement and narrative immersion.
2) A low-performing documentary-style video, characterised by static and informational content with minimal storytelling elements.

This comparative design allowed participants to reflect on differences in emotional resonance, perceived authenticity, and engagement mechanisms. It also enabled the identification of key drivers of narrative transportation and barriers to effective communication. All interviews were conducted in Indonesian to preserve linguistic and cultural authenticity. Data were audio-recorded with participant consent and transcribed verbatim to ensure accuracy and analytical rigor.

### 3.4 Data analysis

Data were analyzed using a Directed Qualitative Content Analysis (DQCA) approach ( Assarroudi *et al*., 2018; Hsieh & Shannon, 2005). This method is particularly suitable when existing theory or prior research can guide the initial coding framework, while still allowing for the emergence of new insights from empirical data. In this study, DQCA was used to validate and extend a predefined theoretical framework consisting of Narrative Transportation, Dark Social, and Institutional Logics. A deductive category matrix was developed as the primary analytical structure, enabling systematic organisation and comparison of data across informants.

At the same time, the analysis remained open to inductive sub-category development, allowing unexpected themes such as specific emotional triggers or organizational bottlenecks to emerge organically from the data. All interview transcripts were imported into ATLAS.ti 9 ( Friese, 2019; Paulus & Lester, 2022) to support: systematic coding, relationship mapping between categories, and transparent documentation of analytical decisions (audit trail). The analytical process followed a structured sequence:

1) Data familiarisation: Repeated reading of transcripts and listening to recordings to understand context and nuance.
2) Development of a deductive coding matrix: Based on the theoretical framework.
3) Coding and categorisation: Assigning meaning units to predefined categories while generating inductive sub-categories.
4) Synthesis and interpretation: Identifying relationships between categories and developing higher-order themes

This process led to the identification of a central phenomenon, institutional friction which reflects the tension between audience expectations for emotionally engaging content and organizational constraints in content production.

### 3.5 Rigor, Reflexivity, and Ethical Considerations

To ensure methodological rigor, this study applies the trustworthiness criteria of credibility, transferability, dependability, and confirmability ( Lincoln & Guba, 1985; Tobin & Begley, 2004).

1) Credibility was achieved through triangulation of data sources, member checking, and prolonged engagement with the research context.
2) Transferability was supported by providing detailed descriptions of participants, context, and the digital health communication environment.
3) Dependability was ensured through a systematic audit trail documenting all research processes and analytical decisions.
4) Confirmability was maintained by ensuring consistency between raw data and interpretations, supported by external review.

Reflexivity was an integral part of the research process. The researcher continuously reflected on their positionality and potential biases through regular peer debriefing sessions with an academic supervisor. This process enhanced analytical transparency and reduced subjectivity. Ethical considerations were strictly adhered to throughout the study. Ethical approval was obtained prior to data collection. All participants provided verbal informed consent, and confidentiality was ensured through anonymisation using alphanumeric codes. The study complies with internationally recognised ethical standards for research involving human participants, including the principles outlined in the Declaration of Helsinki.

## 4. Findings and discussions

This section presents the qualitative findings derived from in-depth stimulated recall interviews, aimed at uncovering the mechanisms shaping the effectiveness of hospital social media communication. In line with the research objectives, the analysis addresses two key issues: (1) how institutional dynamics constrain the implementation of narrative-driven content, and (2) why emotionally engaging content does not consistently translate into observable electronic word-of-mouth (e-WOM).

**Figure 2.**
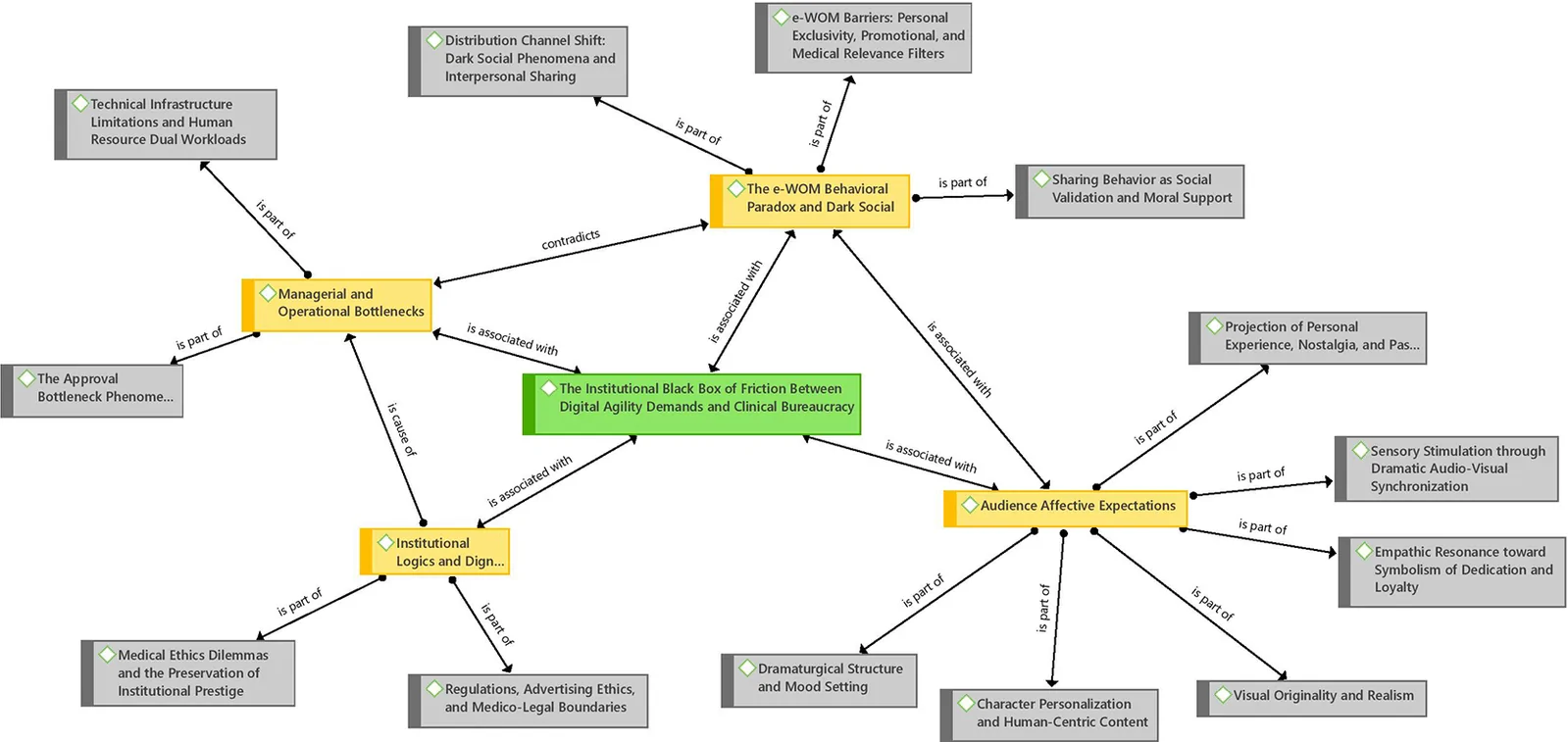
The institutional black box of friction between digital agility demands and clinical bureaucracy.

Consistent with the research design, the findings were developed through a Directed Qualitative Content Analysis (DQCA). The data were mapped onto a predefined deductive framework comprising four primary categories, illustrating the friction between audience expectations and institutional realities. The results indicate that communication effectiveness in healthcare is not solely determined by content characteristics, but rather emerges from the dynamic interaction between audience affective expectations, mediated sharing behaviors, and institutional constraints. To illustrate these relationships, Figure 2 presents an integrative framework synthesizing the four interrelated categories identified in this study.

As shown in Figure 2, audience affective expectations constitute the primary driver of engagement, which subsequently interacts with the e-WOM behavioral paradox shaped by dark social practices. However, these externally driven dynamics are constrained by internal organizational factors, including managerial bottlenecks and broader institutional logics. Notably, the findings reveal a structural tension between the demand for emotionally engaging content and the institutional imperative to preserve professional dignity and credibility.

### 4.1 Audience affective expectations

This category demonstrates that audience engagement is fundamentally governed by affective expectations rather than content characteristics alone.

**Figure 3.**
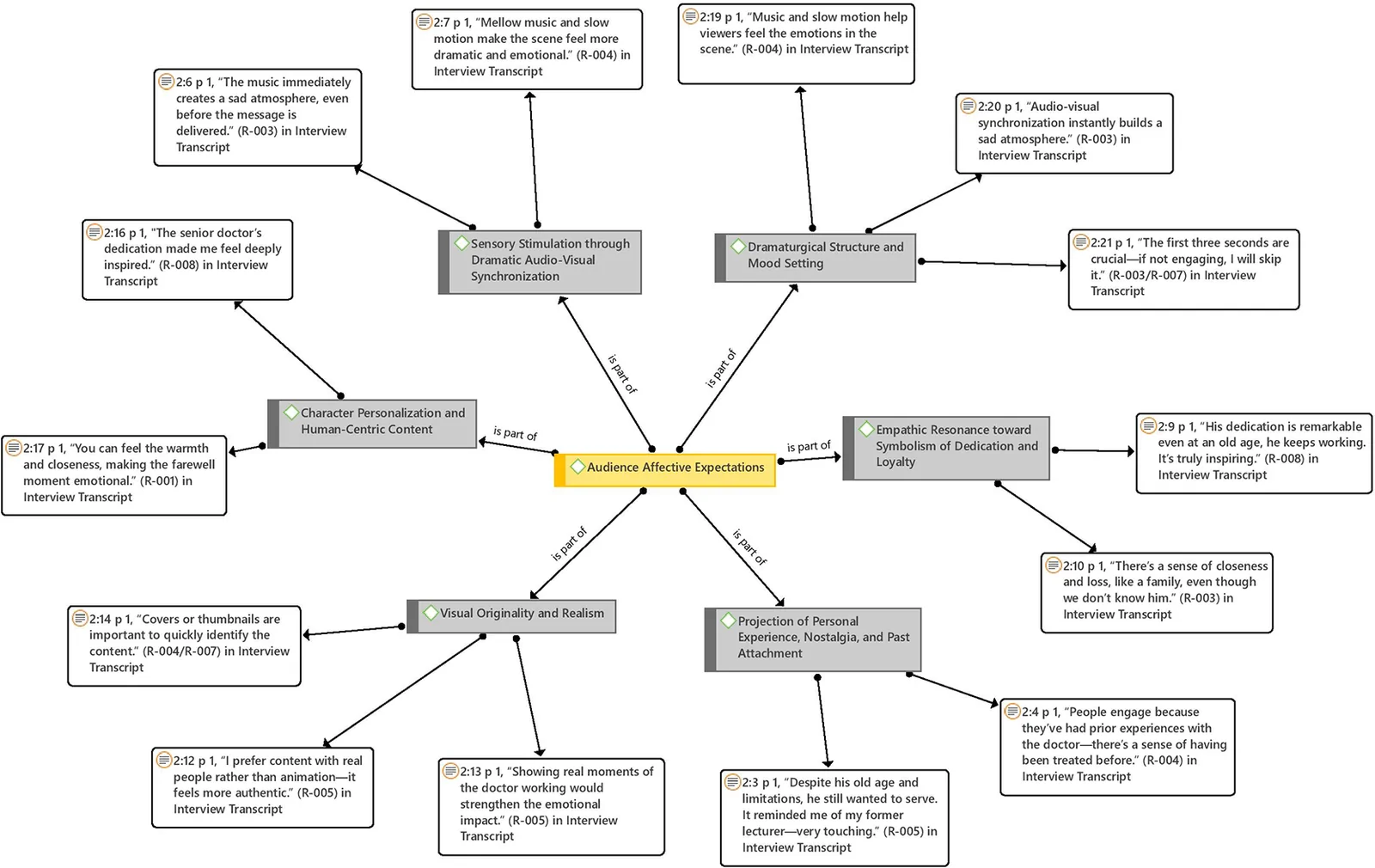
Audience affective expectations.

As illustrated in Figure 3, engagement emerges from an integrated affective system consisting of five interrelated mechanisms: sensory stimulation, dramaturgical structuring, character humanization, visual authenticity, and personal projection. Rather than reflecting abstract preferences, emotional engagement is constructed through concrete viewing experiences. Sensory stimulation particularly through audio-visual synchronization creates an immediate emotional atmosphere that precedes cognitive processing. At the same time, dramaturgical structuring determines whether audiences remain engaged within the critical first seconds of exposure. These findings reinforce the dominance of affective processing in fast-paced digital environments.

Engagement is further intensified through human-centric storytelling. Audiences respond more strongly to personalized portrayals of healthcare professionals, where empathy, dedication, and sacrifice serve as indirect signals of service quality. This is reinforced by visual authenticity, as real-life representations enhance both credibility and emotional resonance. Moreover, emotional responses are amplified through personal projection, allowing audiences to interpret content based on their own experiences and social contexts. These mechanisms converge into empathic resonance, where viewers develop emotional proximity even toward unfamiliar individuals. Overall, this category establishes affective expectations as a central driver of engagement, extending existing literature beyond predominantly cognitive explanations of audience behavior.

The findings reveal that audience engagement in healthcare social media is predominantly driven by affective mechanisms rather than rational-cognitive evaluations. Audiences consistently reject rigid, institution-centric documentation in favor of human-centric storytelling that highlights the dedication and empathy of healthcare professionals. This aligns with Narrative Transportation Theory ( Green & Brock, 2000), suggesting that well-constructed narratives reduce cognitive resistance and foster deep emotional immersion.

In the context of healthcare, which is fundamentally a credence service, clinical quality is notoriously difficult for lay audiences to evaluate objectively ( Ostrom *et al*., 2015). This study provides empirical qualitative evidence supporting the concept of *surrogate evidence* ( Andraus Quintero *et al*., 2025). Audiences use the visual authenticity of dedicated healthcare workers as a heuristic proxy to evaluate the overall credibility and warmth of the institution. Furthermore, the capacity to trigger high-arousal emotions such as awe and inspiration through audio-visual synchronization proves to be a critical catalyst. As Berger and Milkman (2012) and Moriuchi and Gonzalez (2026) posit, emotional arousal, rather than mere positive valence, is the primary engine of digital engagement. This study demonstrates that without intense sensory stimulation, hospital content fails to disrupt the audience’s habitual scrolling behavior.

### 4.2 The e-WOM behavioral paradox and dark social

This category addresses the discrepancy between high affective engagement and low observable public e-WOM. Contrary to linear assumptions that emotional engagement directly leads to sharing behavior, the findings reveal a mediated and selective process.

**Figure 4.**
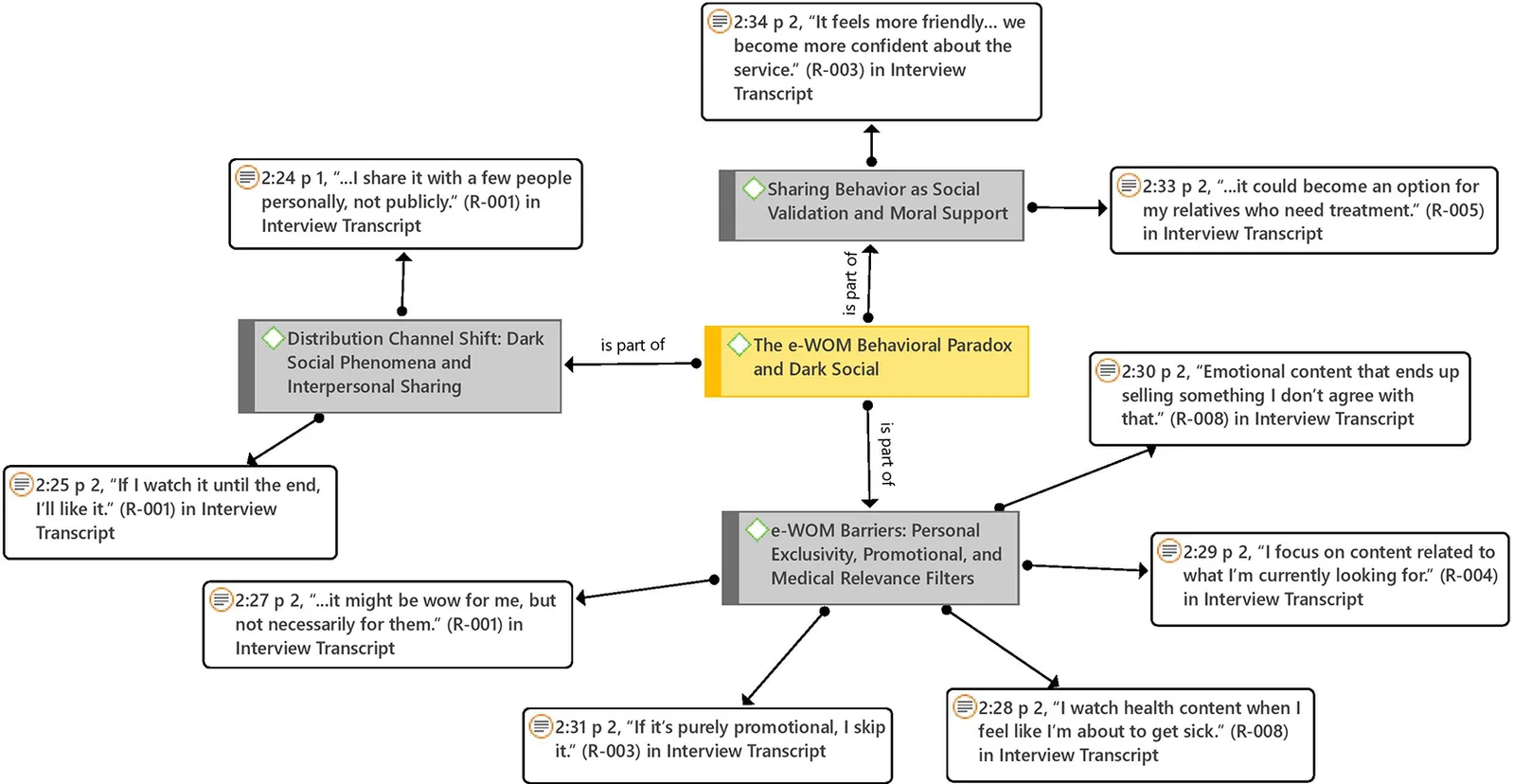
The e-WOM behavioral paradox and dark social.

As illustrated in Figure 4, audience sharing behavior is regulated by three primary filters: personal exclusivity, promotional sensitivity, and situational relevance. The exclusivity filter reflects impression management dynamics, where individuals selectively share content that aligns with their desired online identity. Health-related content, despite its emotional impact, is often perceived as lacking the symbolic value required for public display.

Secondly, audiences demonstrate strong resistance toward overtly promotional content, as such messages may undermine perceived authenticity. Thirdly, sharing decisions are shaped by situational relevance, where content is evaluated based on immediate personal or relational needs rather than habitual engagement patterns. As a consequence, a significant shift occurs from public sharing to private communication channels referred to as Dark Social. Audiences prefer disseminating healthcare information through trusted interpersonal networks (e.g., WhatsApp or direct messaging) due to concerns related to privacy and psychological safety. Importantly, sharing in these spaces is driven by relational motives, such as providing support or recommending services to close contacts, rather than performative self-presentation. These findings demonstrate that the effectiveness of healthcare communication cannot be fully captured through visible engagement metrics, as its impact is often embedded within unobservable private interactions.

A major contribution of this study is the untangling of the engagement-behavior gap. Despite experiencing profound emotional resonance, audiences exhibit a severe reluctance to amplify hospital content through public e-WOM. This behavioral dissonance can be explained through the lens of Impression Management and Social Currency ( Mishra & Singh, 2021). Audiences meticulously curate their public digital personas and often perceive health-related content as lacking the “wow factor” necessary for public broadcast.

Moreover, the episodic nature of health needs and the stigma associated with medical conditions activate a robust psychological defense mechanism ( Jordá *et al*., 2021; Börsting, 2022). Consequently, audiences strategically migrate their sharing behaviors to *Dark Social* private, encrypted interpersonal channels such as WhatsApp or Direct Messages ( RadiumOne, 2016). In these safe spaces, sharing transforms from a performative public act into a genuine gesture of moral support and interpersonal recommendation ( Zomorodpoush *et al*., 2025; Sharma & Sharma, 2025). This finding challenges the traditional paradigm of digital marketing metrics, arguing that the true virality and effectiveness of healthcare communication are often invisible to public social media analytics.

### 4.3 Managerial barriers and operational bottlenecks

This category shifts the focus to internal organizational dynamics, highlighting structural constraints that impede digital agility. The findings reveal that the implementation of narrative-driven content is not merely a creative challenge but is significantly constrained by institutional processes.

**Figure 5.**
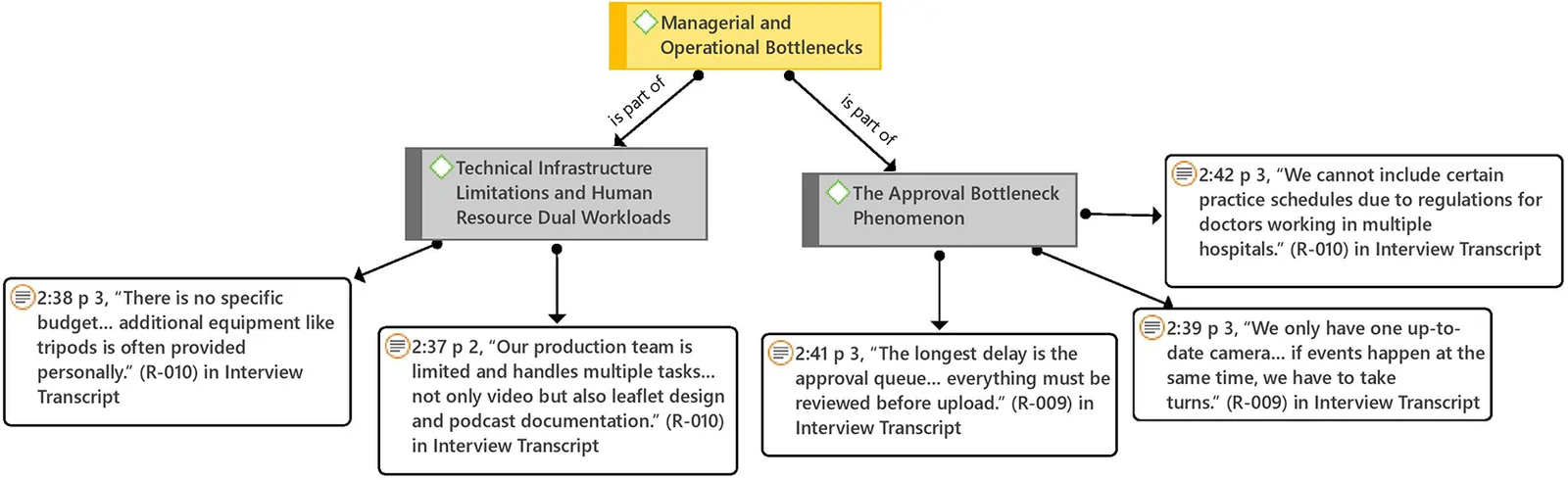
Managerial barriers and operational bottlenecks.

As illustrated in Figure 5, the primary barrier is the approval bottleneck, where content must pass through multiple layers of medical and administrative review. While this process ensures clinical accuracy and risk mitigation, it introduces substantial delays that undermine the timeliness required for effective social media communication.

In addition, the analysis identifies significant limitations in technical infrastructure and human resources. As noted by one informant, content production often relies on personal equipment due to the absence of dedicated budgets (Informant R-010). Furthermore, the presence of dual workloads where digital content creation is treated as a secondary responsibility indicates that social media management is not yet institutionalized as a strategic function. These constraints create a structural misalignment between dynamic audience expectations and rigid organizational capabilities. While audiences demand authentic and responsive content, the institution remains anchored in bureaucratic processes that prioritize caution over agility.

These constraints create a structural misalignment between dynamic audience expectations and rigid organizational capabilities. The institution remains anchored in bureaucratic processes that prioritize caution over agility, creating an internal reality that presents a starkly contrasting picture to the fluid demands of digital media. This qualitative synthesis exposes the ‘Institutional Black Box,’ characterized by chronic resource constraints, dual workloads, and a debilitating approval bottleneck. From a Resource-Based View (RBV) perspective, the lack of dedicated infrastructure and budget for digital content creation paralyzes the creative capabilities of the marketing team. ( Naeem & Ozuem, 2021; Walsh *et al*., 2022).

More fundamentally, this operational friction is rooted in competing Institutional Logics ( Friedland & Alford, 1991; Thornton *et al*., 2012). Hospitals operate under a dominant professional logic that prioritizes risk mitigation, patient privacy, and clinical dignity. The findings demonstrate an intense ethical tension: while algorithms reward sensationalism and trend-jacking, hospital management actively resists these tactics to preserve institutional prestige ( Alhafez *et al*., 2023; de Oliveira Araújo *et al*., 2020). The mandatory pursuit of written informed consent, while legally and ethically imperative, inadvertently strips content of its natural authenticity, creating highly choreographed and sanitized narratives. This structural rigidity explains why hospitals inherently struggle to achieve digital agility, as the imperative to comply with medical regulations constantly throttles creative momentum ( Hügle & Grek, 2023; Udayana *et al*., 2024).

### 4.4 Institutional logics and the preservation of dignity

The final category elaborates on the overarching influence of institutional logics, particularly the tension between digital adaptability and the preservation of professional dignity. The findings indicate that content production is governed not only by creativity but also by strict medico-legal and ethical considerations, including patient privacy, clinical accuracy, and advertising regulations.

**Figure 6.**
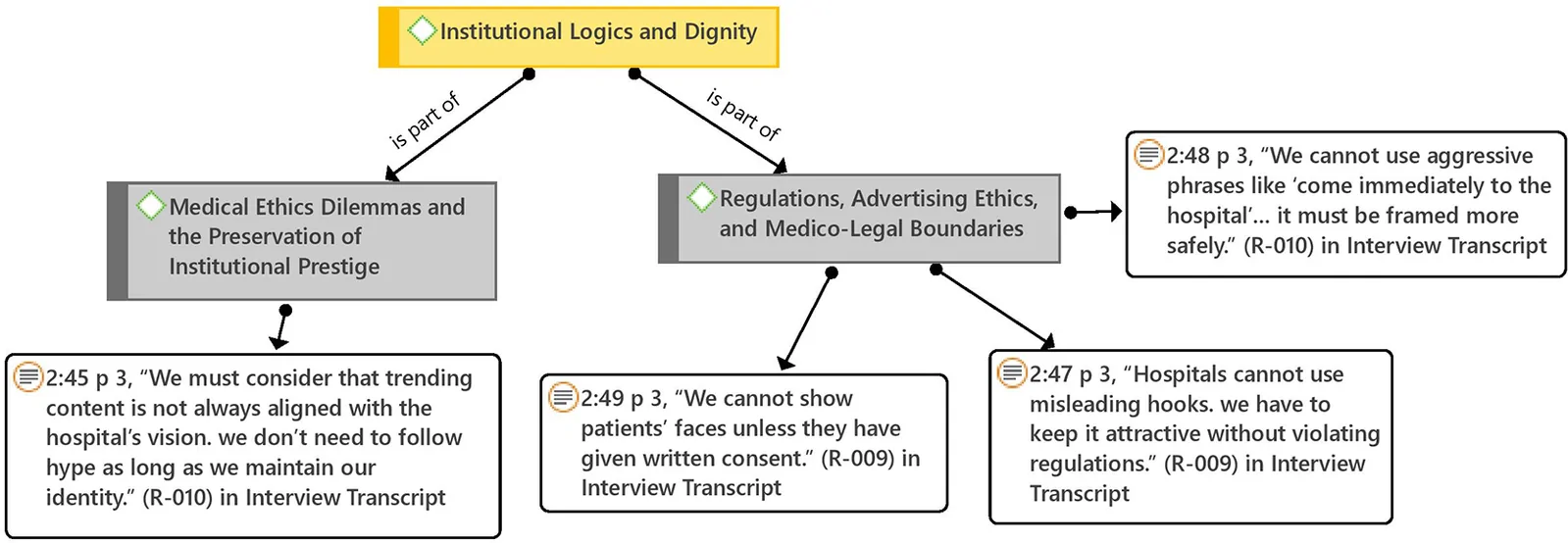
Institutional Logics and the Preservation of Dignity.

As illustrated in Figure 6, these constraints necessitate a “safe framing” of communication, often limiting the use of emotionally provocative or attention-grabbing elements. Beyond formal regulations, there is a strong emphasis on maintaining institutional prestige. Management demonstrates a cautious stance toward adopting popular social media trends due to concerns that such approaches may compromise credibility among key stakeholders.

This reflects the dominance of professional logic, where institutional identity and trust are prioritized over virality or algorithmic visibility. Consequently, emotionally engaging content is often moderated or avoided when it is perceived as potentially undermining clinical authority. This category reveals a fundamental paradox: although narrative and emotional content are empirically effective in engaging audiences, their implementation is constrained by institutional norms that prioritize dignity, trust, and compliance. As a result, storytelling strategies are frequently diluted within the hospital’s digital ecosystem despite their recognized value.

### 4.5 Practical implications: The Quality Control (QC) Checklist

To reconcile the fundamental asymmetry between fluid audience expectations and rigid organizational capabilities, this study conceptualized and validated a specialized Quality Control (QC) Checklist. In healthcare management, the absence of standardized creative workflows results in a high *Cost of Poor Quality* (COPQ), characterized by wasted man-hours on rejected or ineffective content ( Choi *et al*., 2025).

The QC Checklist acts as an institutional nexus, translating tacit ethical boundaries and aesthetic preferences into explicit, pre-approved guidelines ( Nothacker *et al*., 2025). By instituting “filtering gates” based on narrative structure, affective stimulation, social currency, and regulatory compliance, hospitals can bypass repetitive bureaucratic reviews. This mechanism effectively reduces the approval turnaround time and alleviates operational burnout ( Hassmann *et al*., 2025), enabling hospitals to execute narrative strategies with digital agility without compromising their medico-legal integrity.

## 5. Conclusion and implication

This study concludes that the implementation of narrative communication strategies in hospital social media is a complex and non-linear process, constrained by the structural friction between digital agility and clinical bureaucracy. Through the lens of Directed Qualitative Content Analysis (DQCA), the findings reveal that while narrative-driven content is highly effective in fostering emotional immersion and reducing cognitive resistance, its routine adoption is systematically throttled by deep-seated institutional dynamics.

Regarding the first research question, this study identifies that dominant institutional logics prioritizing risk mitigation, clinical accuracy, and the preservation of professional dignity create significant approval bottlenecks. These structural constraints, compounded by limited technical infrastructure and dual workloads of the creative staff, lead to a loss of “real-time” relevance, as content often undergoes multiple layers of administrative and medical review before publication. This institutional rigidity explains why hospitals, despite recognizing the potential of storytelling, frequently default to formalized and ceremonial documentation that fails to resonate with audience expectations.

In addressing the second research question, this research untangles the paradox between high affective engagement and low observable public e-WOM. The analysis demonstrates that audiences perform a rigorous mental filtration based on social currency and medical privacy filters. Rather than amplifying healthcare messages in public digital spaces, audiences strategically shift their sharing behaviors toward “Dark Social” channels such as private messaging applications which provide a safe space for interpersonal support and genuine recommendations. This indicates that the true effectiveness of hospital digital communication is often embedded within unobservable private dialogues rather than public metrics.

Theoretically, this study extends the application of Narrative Transportation Theory and Institutional Logics within the high-trust healthcare sector. It provides empirical qualitative evidence that in digital environments, affective transmission is more dominant than rational-cognitive evaluation in determining audience engagement. Practically, the study conceptualizes a Strategic Quality Control (QC) Checklist as an institutional nexus to reconcile the fundamental asymmetry between digital agility and bureaucratic constraints. The implementation of this standardized framework allows hospitals to streamline approval processes and minimize operational burnout while maintaining strict adherence to medical ethics and patient privacy.

## 6. Limitations and future research

While this study provides novel insights into the institutional and psychological mechanisms governing hospital social media communication, several limitations must be acknowledged to contextualize the findings and guide future inquiries.

First, the exploration of electronic word-of-mouth (e-WOM) and Dark Social relied predominantly on self-reported behavioral intentions and retrospective accounts elicited during stimulated recall interviews, rather than direct observation of digital footprints. Because Dark Social sharing occurs within encrypted, private channels, empirically capturing the actual volume of distributed content remains a methodological challenge. Future research should consider employing quasi-experimental field studies or utilizing digital tracking software such as UTM (Urchin Tracking Module) parameters to quantitatively measure the actual web traffic generated through these private interpersonal networks. Transitioning the focus from sharing *intentions* to *actual behavior* will provide a more precise valuation of narrative content effectiveness.

Second, this study is contextually bounded by its focus on a single private hospital in Indonesia. The institutional logics and bureaucratic bottlenecks identified while reflective of high-trust healthcare environments may manifest differently in other organizational settings. Public or government-owned hospitals, which typically operate under more rigid, highly bureaucratic hierarchical structures, may experience different ethical tensions and approval dynamics compared to private institutions. Therefore, future studies are highly encouraged to conduct comparative institutional analyses between private and public healthcare facilities to examine how varying degrees of bureaucratic rigidity impact digital agility.

Finally, to further unpack the “Institutional Black Box,” future research could explore additional internal organizational dimensions. Investigating factors such as organizational culture, leadership styles, and overall digital readiness as underlying structural influences would provide a deeper understanding of the internal contingencies that either facilitate or constrain the successful execution of narrative-driven communication strategies in the healthcare sector.

## Ethics and consent

Ethical approval was granted by the Medical and Health Research Ethics Committee (MHREC) Faculty of Medicine, Public Health and Nursing Universitas Gadjah Mada - Dr. Sardjito General Hospital (Approval Number: KE/FK/0160/EC/2026). Verbal informed consent for participation in the study and for the publication of the participants’ de-identified responses was obtained from all participants prior to the interviews. This verbal consent process was witnessed and recorded (via audio or video recording for remote sessions), as approved by the Medical and Health Research Ethics Committee (MHREC) Faculty of Medicine, Public Health and Nursing Universitas Gadjah Mada - Dr. Sardjito General Hospital under the study protocol.

## Data Availability

Zenodo: Dataset for: The Institutional Black Box of Friction Between Digital Agility Demands and Clinical Bureaucracy in Hospital Social Media Management. DOI: https://doi.org/10.5281/zenodo.19914645 ( Renaldi *et al*., 2026). This project contains the following underlying data:
•
Underlying_Data_Qualitative_Coding_Matrix.xlsx (Coding matrix of audience and management interview transcripts). Zenodo: Dataset for: The Institutional Black Box of Friction Between Digital Agility Demands and Clinical Bureaucracy in Hospital Social Media Management. DOI: https://doi.org/10.5281/zenodo.19914645 ( Renaldi *et al*., 2026). This project contains the following extended data:
•
Extended_Data_Instrument_1_Hospital_Narrative_QC_Checklist.pdf (Quality Control Checklist validation instrument). •
Extended_Data_Instrument_2_Interview_Protocols_Audience_and_Management.pdf (Stimulated recall interview guidelines). Data are available under the terms of the Creative Commons Attribution 4.0 International license (CC-BY 4.0). This study was reported in accordance with the Consolidated criteria for reporting qualitative research (COREQ) guidelines.

## References

[ref3] Alhafez L Rubio-Rico L Diez-Bosch M : Healthcare professionals’ editorial opinions on communicating with the public: shifting social media hesitancies. *Humanit Soc Sci Commun.* 2023;10(1). 10.1057/s41599-023-01820-w

[ref4] Andraus Quintero CE Vega Mendoza DL Esquivel Garcia R : The impact of social media on healthcare professionals’ branding. *Salud Ciencia Y Tecnologia.* 2025;5:1539. 10.56294/saludcyt20251539

[ref5] Assarroudi A Nabavi FH Armat MR : Directed qualitative content analysis: The description and elaboration of its applications. *Nurs. Open.* 2018;5(1):158–166. 10.1002/nop2.123 34394406 PMC7932246

[ref6] Ayuningtyas D : Digital marketing's impact to hospital visits during COVID-19: A systematic review. *Syntax Literate: Jurnal Ilmiah Indonesia.* 2022;7(5). 10.36418/syntax-literate.v7i5.7223

[ref7] Bansal G Zahedi FM Gefen D : The impact of personal dispositions on information sensitivity, privacy concern and trust in disclosing health information online. *Decis. Support. Syst.* 2010;49(2):138–150.

[ref8] Beldad A Hegner S : Expanding the technology acceptance model with the inclusion of trust, social influence, and health consciousness: The case of wearable fitness trackers. *Int. J. Hum. Comput. Interact.* 2018;34(9):882–894. 10.1080/10447318.2017.1420038

[ref9] Berger J : Word of mouth and interpersonal communication: A review and directions for future research. *J. Consum. Psychol.* 2014;24(4):586–607. 10.1016/j.jcps.2014.05.002

[ref10] Berger J Milkman KL : What makes online content viral?. *J. Mark. Res.* 2012;49:192–205. 10.1509/jmr.10.0353

[ref11] Börsting J : Online Health Information Exchange and Privacy. *International Encyclopedia of Health Communication.* 2022. 10.1002/9781119678816.iehc0994

[ref12] Choi H Jeong I Cheon J : Application of sigma-based quality control rules for the efficiency of internal quality control. *Practical Laboratory Medicine.* 2025;47:e00501. 10.1016/j.plabm.2025.e00501 40978770 PMC12446610

[ref13] Oliveira Araújo EP de Paula CPA de Silva Neto JR da : The meme as an institutional marketing strategy in digital social medias. *Media E Jornalismo.* 2020;20(36):73–91. 10.14195/2183-5462_36_4

[ref14] Denzin NK : *The research act: A theoretical introduction to sociological methods.* McGraw-Hill;1978.

[ref15] Dessart L Veloutsou C Morgan-Thomas A : Consumer engagement in online brand communities: A social media perspective. *J. Prod. Brand. Manag.* 2015;24(1):28–42. 10.1108/JPBM-06-2014-0635

[ref16] Dinas Kesehatan Kota Yogyakarta: *Profil Kesehatan Kota Yogyakarta Tahun 2023.* Dinas Kesehatan Kota Yogyakarta;2024.

[ref17] Dzakiyya N Hijrah Hati S : The impact of social media marketing on hospital brand equity. *J. Health Manag.* 2024;12(2):45–58.

[ref18] Escalas JE : Narrative processing: Building consumer connections to brands. *J. Consum. Psychol.* 2004;14(1–2):168–180. 10.1207/s15327663jcp1401&2_19

[ref19] Friedland R Alford RR : Bringing society back in: symbols, practices, and institutional contradictions. Powell WW DiMaggio PJ , editors. *The New Institutionalism in Organizational Analysis.* University of Chicago Press;1991; pp.232–263.

[ref20] Friese S : *Qualitative data analysis with ATLAS.ti.* Sage Publications; 3rd ed 2019.

[ref21] Green MC Brock TC : The role of transportation in the persuasiveness of public narratives. *J. Pers. Soc. Psychol.* 2000;79(5):701–721. 10.1037/0022-3514.79.5.701 11079236

[ref22] Griffioen N Van Rooij MMJW Lichtwarck-Aschoff A : A stimulated recall method for the improved assessment of quantity and quality of social media use. *J. Med. Internet Res.* 2020;22(1):e16929. 10.2196/15529 PMC7013654 32012075

[ref23] Guba EG Lincoln YS : Competing paradigms in qualitative research. Denzin NK Lincoln YS , editors. *Handbook of qualitative research.* Sage Publications;1994;105–117.

[ref24] Hassmann J Kröner S Niemöller L : Can digital smart workflows in a hospital free staff from documentation and administrative work? *Studies in Health Technology and Informatics.* 2025; Vol.332. 10.3233/SHTI251509 41041759

[ref25] Hennink M Kaiser B : Sample sizes for saturation in qualitative research: A systematic review of empirical tests. *Soc. Sci. Med.* 2022;292:114523. 10.1016/j.socscimed.2021.114523 34785096

[ref26] Hennink M Kaiser B Marconi VC : Code saturation versus meaning saturation: How many interviews are enough?. *Qual. Health Res.* 2017;27(4):591–608. 10.1177/1049732316665344 27670770 PMC9359070

[ref27] Hollebeek LD Glynn MS Brodie RJ : Consumer brand engagement in social media: Conceptualization, scale development and validation. *J. Interact. Mark.* 2014;28(2):149–165. 10.1016/j.intmar.2013.12.002

[ref28] Hsieh H-F Shannon SE : Three approaches to qualitative content analysis. *Qual. Health Res.* 2005;15(9):1277–1288. 10.1177/1049732305276687 16204405

[ref29] Hügle T Grek V : Digital transformation of an academic hospital department: A case study on strategic planning using the balanced scorecard. *PLoS Digit Health.* 2023;2(11). 10.1371/journal.pdig.0000385 PMC10656018 37976272

[ref30] Jordá A : Healthcare professionals' use of social media: A systematic review. *J. Med. Internet Res.* 2021.

[ref31] Kreps GL Neuhauser L : New directions in eHealth communication: Opportunities and challenges. *Patient Educ. Couns.* 2010;78(3):329–336. 10.1016/j.pec.2010.01.013 20202779

[ref32] Kurniawan A Berlianto MP : Pengaruh social media marketing terhadap kepercayaan dan minat kunjungan pasien rumah sakit. *J Manaj Kesehat Indones.* 2022;10(1):12–25.

[ref33] Lincoln YS Guba EG : *Naturalistic inquiry.* Sage Publications;1985.

[ref34] Lyle J : Stimulated recall: A report on its use as a means of exploring a coach’s decision-making process. *Int J Appl Sports Sci.* 2003;15(1):24–37. 10.1080/0141192032000137349

[ref35] Madrigal AC : *Dark social: We have the whole history of the web wrong.* The Atlantic;2012.

[ref36] Mishra Y Singh A : Identifying the factors of social currency for social media marketing strategy. *South Asian Journal of Business Studies.* 2021;10(3):305–321. 10.1108/SAJBS-01-2020-0022

[ref37] Moriuchi E Gonzalez C : The role of emotional arousal in digital engagement: A cross-platform analysis. *J. Interact. Mark.* 2026.

[ref38] Naeem M Ozuem W : Exploring the use of social media sites for health professionals’ engagement and productivity in public sector hospitals. *Empl. Relat.* 2021;43(5):1029–1051. 10.1108/ER-08-2020-0391

[ref39] Nothacker M : Digitalisation of the guideline registry of the Association of Scientific Medical Societies in Germany. *BMJ Open.* 2025;15(11). 10.1136/bmjopen-2024-095294 PMC12587974 41248342

[ref40] Ostrom AL Parasuraman A Bowen DE : Service research priorities in a rapidly changing context. *J. Serv. Res.* 2015;18(2):127–159. 10.1177/1094670515576315

[ref41] Patton MQ : *Qualitative research & evaluation methods: Integrating theory and practice.* Sage Publications; 4th ed 2015.

[ref42] Paulus TM Lester JN : *Doing qualitative research in a digital world.* Sage Publications;2022.

[ref43] RadiumOne: *The dark side of mobile sharing: How dark social is driving digital engagement.* RadiumOne Research;2016.

[ref44] Reay T Hinings CR : Managing the rivalry of competing institutional logics. *Organ. Stud.* 2009;30(6):629–652. 10.1177/0170840609104803

[ref45] Renaldi US Rosa R Wulandari EE : Dataset for: **The Institutional Black Box of Friction Between Digital Agility Demands and Clinical Bureaucracy in Hospital Social Media Management** **[Data set]**. *Zenodo.* 2026. 10.5281/zenodo.19914645

[ref46] Saunders MNK Lewis P Thornhill A : *Research methods for business students.* Pearson Education; 8th ed 2019.

[ref47] Scott WR : *Institutions and organizations: Ideas, interests, and identities.* Sage Publications;2014.

[ref48] Sharma A Sharma BK : Unveiling the power of dark social: Leveraging user-generated content for brand advocacy, social proof, and user engagement. *J. Mark. Commun.* 2025;1–30. 10.1080/13527266.2025.2547705

[ref49] Thornton PH Ocasio W Lounsbury M : *The Institutional Logics Perspective: A New Approach to Culture, Structure, and Process.* Oxford University Press;2012. 10.1093/acprof:oso/9780199601936.001.0001

[ref50] Tobin GA Begley CM : Methodological rigour within a qualitative framework. *J. Adv. Nurs.* 2004;48(4):388–396. 10.1111/j.1365-2648.2004.03207.x 15500533

[ref51] Udayana AGB : Digital marketing innovation and post-pandemic recovery: a comparative study of private hospital visitation in Bali. *Econ Ann XXI.* 2024;212(11–12):33–38. 10.21003/ea.V212-06

[ref52] Walsh L : Social Media as a Tool for Consumer Engagement in Hospital Quality Improvement and Service Design: Barriers and Enablers for Implementation. *Int. J. Health Policy Manag.* 2022;11(10):2287–2298. 10.34172/ijhpm.2021.151 34814682 PMC9808274

[ref53] World Health Organization: *Second round of the national pulse survey on continuity of essential health services during the COVID-19 pandemic.* WHO;2021.

[ref54] Zhao Y Zhang J Liu Y : The impact of trust on consumers' online health information seeking behavior. *J. Retail. Consum. Serv.* 2020;55:102115. 10.1016/j.jretconser.2020.102115

[ref55] Zomorodpoush T Fahimnia F Noruzi A : The effect of perceived risk and benefit on the search and sharing of health information by social network users. *Iran J Inf Process Manage.* 2025;40(3):931–964. 10.22034/jipm.2024.2036084.1693

