# Supplementary material for: The Institutional Black Box of Friction Between Digital Agility Demands and Clinical Bureaucracy in Hospital Social Media Management
Source: F1000Res. 2026 Jul 3;15:820. Originally published 2026 May 27. [Version 2] doi: 10.12688/f1000research.180694.2 (PMC13550901; doi:10.12688/f1000research.180694.2)
Supplement: Peer review history

# Reviewer report: Reviewer response for version 2

**DOI:** 10.5256/f1000research.204810.r498216

**Published:** 2026-07-06

**Authors:** Iwona Czerska

**Affiliations:** 1 Wroclaw University of Economics and Business, Wroclaw, Poland

Thank you for the revised manuscript and for the detailed response to my previous comments. I have reviewed Version 2 and the authors’ response, and I consider that my main reservations have been adequately addressed. The revised article now provides a clearer justification of sampling and saturation, adds important details on collaborative coding, member checking, peer debriefing, and audit trail procedures, clarifies the self-reported basis for the dark social interpretation, moderates claims about the QC checklist, and improves the data availability and limitations sections. These revisions strengthen the methodological transparency, analytical auditability, and interpretive caution of the manuscript.

 I have no further substantive comments. As a minor editorial point, I would only suggest ensuring fully consistent wording around the QC checklist, avoiding any implication that it has already been empirically validated unless such evidence is provided. Overall, I consider the revised article acceptable.

Is the work clearly and accurately presented and does it cite the current literature?

Partly

If applicable, is the statistical analysis and its interpretation appropriate?

Not applicable

Are all the source data underlying the results available to ensure full reproducibility?

Partly

Is the study design appropriate and is the work technically sound?

Partly

Are the conclusions drawn adequately supported by the results?

Partly

Are sufficient details of methods and analysis provided to allow replication by others?

Partly

Reviewer Expertise:

Healthcare management, healthcare marketing, digital health communication, social media marketing, patient engagement, and qualitative research

I confirm that I have read this submission and believe that I have an appropriate level of expertise to confirm that it is of an acceptable scientific standard.

# Reviewer report: Reviewer response for version 1

**DOI:** 10.5256/f1000research.199321.r490095

**Published:** 2026-06-19

**Authors:** Nugzar Todua

**Affiliations:** 1 Ivane Javakhishvili Tbilisi State University, Tbilisi, Georgia

The work is interesting, although we have a few comments about it.

 1. Insufficient Organizational Representation. The study’s central argument concerns institutional dynamics and bureaucratic constraints within hospitals; however, the internal perspective is based on only two management informants. This limited representation raises concerns regarding the credibility and completeness of the findings. Key stakeholders such as legal/compliance officers, physicians, senior administrators, and social media managers are absent. The authors should justify why these perspectives were excluded or acknowledge more explicitly how this limitation may have shaped the conclusions.

 2. Weak Evidence for the “Dark Social” Explanation. The manuscript presents dark social as a major explanation for the engagement–e-WOM gap, yet the evidence relies exclusively on participants’ self-reported perceptions and retrospective accounts. No behavioral data, platform analytics, or digital trace evidence are provided to substantiate these claims. Consequently, the conclusions regarding dark social appear speculative and should be reframed as plausible interpretations rather than demonstrated findings.

 3. Overextension of Theoretical Claims. The study claims to extend Narrative Transportation Theory and Institutional Logics in healthcare communication. However, the empirical evidence is derived from a single hospital case and a small qualitative sample. The theoretical contribution is therefore overstated. The authors should clarify whether the proposed framework is exploratory and context-specific rather than presenting it as a broadly applicable theoretical advancement.

 4. Limited Methodological Transparency. The coding process lacks sufficient detail. The manuscript does not clearly explain who conducted the coding, whether intercoder agreement was assessed, how discrepancies were resolved, or how final themes emerged from the data. Greater transparency is necessary to evaluate the rigor, dependability, and confirmability of the qualitative analysis.

 5. Practical Recommendations Are Not Adequately Validated. The proposed Quality Control (QC) Checklist is presented as a solution to reduce approval delays and improve digital agility. However, no empirical evaluation of the checklist’s effectiveness is reported. Without implementation or outcome data, these claims are premature and should be repositioned as preliminary recommendations for future testing rather than evidence-based managerial interventions.

 Considering the above comments, I think the paper can be indexed.

Is the work clearly and accurately presented and does it cite the current literature?

Yes

If applicable, is the statistical analysis and its interpretation appropriate?

Yes

Are all the source data underlying the results available to ensure full reproducibility?

Yes

Is the study design appropriate and is the work technically sound?

Yes

Are the conclusions drawn adequately supported by the results?

Yes

Are sufficient details of methods and analysis provided to allow replication by others?

Partly

Reviewer Expertise:

Marketing

I confirm that I have read this submission and believe that I have an appropriate level of expertise to confirm that it is of an acceptable scientific standard.

# Response

**Published:** 2026-06-24

**Authors:** Renaldi -

**Affiliations:** Health Policy and Management, Gadjah Mada University, Yogyakarta, Special Region of Yogyakarta, Indonesia

Dear Prof. Nugzar Todua,

 Thank you for your constructive feedback and for awarding our manuscript an 'Approved' status. We have carefully revised the manuscript to address all your comments:

**1. Insufficient Organizational Representation:** We revised Section 3.2 to explain our key informant approach for the 2 management actors. We also expanded Section 6 (Limitations) to explicitly acknowledge the absence of broader stakeholders (legal, medical leadership).

**2. Weak Evidence for "Dark Social":** We accept this critique. We reframed the findings (Sections 4.2 and 5) to state that the shift toward dark social is a "plausible interpretation" based on self-reported intentions, rather than measured digital trace evidence.

**3. Overextension of Theoretical Claims:** We revised the Conclusion (Section 5) to clarify that our theoretical contribution is an exploratory, context-specific framework bounded by the single-hospital setting.

**4. Limited Methodological Transparency:** We substantially enriched Sections 3.4 and 3.5, detailing the collaborative coding process using ATLAS.ti and clarifying our synthesized member-checking and audit trail procedures.

**5. Practical Recommendations:** We have tempered our claims regarding the Quality Control (QC) Checklist in Section 4.5. We now explicitly position the checklist as a preliminary recommendation that requires future empirical testing.

# Reviewer report: Reviewer response for version 1

**DOI:** 10.5256/f1000research.199321.r490096

**Published:** 2026-06-08

**Authors:** Iwona Czerska

**Affiliations:** 1 Wroclaw University of Economics and Business, Wroclaw, Poland

The manuscript addresses an important and timely topic: the tension between the need for agile, emotionally engaging digital communication and the bureaucratic, risk-averse logic of hospital organisations. The article focuses on hospital social media management, particularly narrative content, audience engagement, electronic word of mouth, and the role of “dark social”. The authors use a qualitative interpretivist design, stimulated recall interviews, Directed Qualitative Content Analysis, and ATLAS.ti to explore both audience and internal management perspectives. The study identifies four main areas: audience affective expectations, the e-WOM/dark social paradox, managerial and operational bottlenecks, and institutional logics related to dignity, risk, and compliance.

 Overall, the article has a relevant research problem, a promising conceptual framing, and useful practical implications for hospital communication management. The combination of narrative transportation, institutional logics, and dark social is potentially valuable. However, several points require revision before the article can be considered fully scientifically sound. Most importantly, the authors should strengthen methodological transparency, moderate some conclusions, clarify the source data, and improve the critical integration of the literature.

**Main strengths**

 The study addresses a practically significant issue for healthcare organisations: how hospitals can communicate in a more human, narrative, and engaging way without violating professional, ethical, and reputational constraints. The topic is relevant in the post-pandemic context, where digital trust and online communication have become important elements of patient–institution relationships.

 The qualitative design is generally appropriate for exploring institutional tensions, audience interpretations, and hidden or semi-hidden sharing practices. The use of stimulated recall interviews is appropriate because it allows participants to respond to concrete social media stimuli rather than discussing only abstract preferences. The inclusion of both audience members and internal management representatives is also a strength, as it helps connect the reception of content with the organisational processes behind its production.

 The article also makes a useful conceptual contribution by showing that high emotional engagement does not necessarily translate into publicly visible e-WOM. The discussion of dark social is especially relevant in healthcare, where privacy concerns and the sensitivity of medical information may shift communication into private channels.

**Points that must be addressed to make the article scientifically sound**

 First, the authors should provide a much more precise description of the sampling procedure and sample adequacy. The study is based on 10 informants, including 8 audience members and only 2 internal management representatives. This may be acceptable for an exploratory qualitative study. Still, the authors need to explain more clearly how participants were recruited, why this number was considered sufficient, and whether thematic saturation or information power was assessed. The internal organisational side of the study is particularly important for the article’s argument, but it currently relies on a very limited number of management informants. The authors should either justify this limitation more strongly or include additional internal participants representing different organisational levels, such as social media staff, compliance/legal staff, medical leadership, marketing/public relations, and hospital management.

 Second, the analysis procedure needs to be described in greater operational detail. The authors state that Directed Qualitative Content Analysis was used and that deductive and inductive coding were combined. Still, the manuscript should clarify who performed the coding, whether more than one coder was involved, how coding disagreements were resolved, how categories were refined, and how the final themes were derived from the empirical material. The authors should also describe how member checking, peer debriefing, external review, and audit trail procedures were implemented, rather than merely stating that they were used. This is necessary to strengthen credibility, dependability, and confirmability.

 Third, the conclusions about dark social should be moderated. The concept is useful and plausible, but the empirical evidence appears to rely mainly on self-reported intentions and retrospective accounts rather than direct observation of private sharing behaviour. Therefore, the authors should avoid wording that implies that dark social activity was directly measured. A more accurate formulation would be that the findings suggest a tendency for healthcare-related content to be shared privately rather than publicly. The authors themselves acknowledge that direct observation of digital footprints was not conducted and that dark social remains difficult to measure empirically.

 Fourth, the practical claims concerning the Quality Control checklist should be presented more cautiously. The checklist may be a useful managerial tool, but the manuscript should clearly explain how it was developed, validated, and evaluated. At present, claims that the checklist can reduce approval time, alleviate operational burnout, or improve agility appear stronger than the evidence allows. Unless the authors provide stronger validation data, these statements should be reframed as practical propositions or recommendations for future testing.

 Fifth, the data availability statement should be clarified. The manuscript reports that the underlying data and extended data are deposited in Zenodo, including a qualitative coding matrix, a Quality Control Checklist validation instrument, and stimulated recall interview protocols. This is positive. However, it remains unclear whether full anonymised transcripts are available. If full transcripts cannot be shared for ethical or confidentiality reasons, the authors should state this explicitly and explain the level of data access available.

 Sixth, the literature review should be revised to provide a stronger critical synthesis. The manuscript cites relevant literature on narrative transportation, institutional logics, healthcare communication, e-WOM, and dark social, but some parts are descriptive and repetitive. The authors should more clearly explain what is already known, what remains insufficiently understood, and how this study specifically advances the literature. They should also ensure that all recent references are relevant, reliable, and directly connected to the argument.

**Additional comments and suggestions**

 The manuscript would benefit from clearer differentiation between empirical findings, interpretation, and managerial recommendations. Some passages move quickly from qualitative observations to broad theoretical or practical claims. The authors should consistently mark which claims are directly supported by the interview data and which are conceptual extensions.

 The single-hospital context should be emphasised more strongly throughout the discussion and conclusion. The findings are valuable as an in-depth case study, but they should not be generalised to all hospitals without caution. The authors should use language such as “in this case”, “within the studied hospital”, or “the findings suggest” rather than broader claims about hospitals in general.

 The manuscript also needs improvements in language and formatting. Some terms and headings are inconsistent, and there are minor errors or awkward expressions. The figures are conceptually useful but visually dense; they should be simplified or explained more clearly in the text. Tables and figures should be checked for spelling, readability, and consistency.

**Expanded answers to the peer review questions**

 The work is **partly** clearly and accurately presented, and it does cite current literature. The topic is well introduced, and the conceptual framework is relevant. However, the literature review should be more critically synthesised, and some arguments should be made more precise.

 The study design is **partly** appropriate and technically sound. The qualitative approach fits the research questions, but the sample is small, the internal management perspective is underrepresented, and the authors need to provide stronger justification for the sample's adequacy and analytical rigour.

 The methods and analysis are described sufficiently. The manuscript provides useful information on stimulated recall interviews, DQCA, ATLAS.ti, coding, triangulation, and ethics, but it does not yet provide sufficient operational detail to allow full qualitative auditability.

 The statistical analysis is **not applicable** because the study is qualitative and does not use inferential statistical testing.

 The source data are **partly** available. The coding matrix and research instruments are reportedly available, but the status of the full anonymised transcripts or the raw qualitative material should be clarified.

 The results partly support the conclusions. The main conclusions are plausible and generally aligned with the findings, but claims about dark social, virality, checklist effectiveness, and general applicability should be moderated or supported with additional evidence.

**Overall assessment**

 The article has clear potential and addresses an important problem in healthcare communication and hospital management. However, before it can be considered fully scientifically sound, the authors should revise the methodological description, strengthen transparency of the analysis, clarify data availability, moderate overly extended conclusions, and improve the critical integration of the literature. My overall recommendation would be **approval with reservations after substantial revision**.

Is the work clearly and accurately presented and does it cite the current literature?

Partly

If applicable, is the statistical analysis and its interpretation appropriate?

Not applicable

Are all the source data underlying the results available to ensure full reproducibility?

Partly

Is the study design appropriate and is the work technically sound?

Partly

Are the conclusions drawn adequately supported by the results?

Partly

Are sufficient details of methods and analysis provided to allow replication by others?

Partly

Reviewer Expertise:

Healthcare management, healthcare marketing, digital health communication, social media marketing, patient engagement, and qualitative research

I confirm that I have read this submission and believe that I have an appropriate level of expertise to confirm that it is of an acceptable scientific standard, however I have significant reservations, as outlined above.

## References

[1] : Consolidated criteria for reporting qualitative research (COREQ): a 32-item checklist for interviews and focus groups. *International Journal for Quality in Health Care* .2007;19(6) : 10.1093/intqhc/mzm042 349-357 10.1093/intqhc/mzm042 17872937

# Response

**Published:** 2026-06-24

**Authors:** Renaldi -

**Affiliations:** Health Policy and Management, Gadjah Mada University, Yogyakarta, Special Region of Yogyakarta, Indonesia

Dear Dr. Iwona Czerska,

 Thank you for your highly constructive feedback. Your insights have been instrumental in refining the precision of our paper. We have addressed all your points as follows:

**1. Sampling & Representation:** We revised Section 3.2 to detail our purposive sampling, explaining thematic saturation at the 8 ^th^ audience informant, and key informant selection for the 2 management actors. We added a new paragraph in Section 6 acknowledging the underrepresentation of broader stakeholders.

**2. Analytical Transparency:** We revised Sections 3.4 and 3.5, detailing the collaborative coding process using ATLAS.ti to reach team consensus, our synthesized member checking approach (Birt et al., 2016), and our coding matrix audit trail.

**3. Dark Social Claims:** We reframed our conclusion (Sections 4.2 and 5) to state that dark social is a "plausible interpretation" based on self-reported intentions, rather than measured digital footprints.

**4. QC Checklist:** We tempered claims in Section 4.5, positioning the checklist as a preliminary practical recommendation for future testing.

**5. Data Availability:** We explicitly stated in the Data Availability section that full un-redacted transcripts cannot be shared publicly due to strict ethical constraints, directing readers to the matrix in Zenodo and providing a conditional access mechanism.

**6. Literature & Context:** We added a synthesizing paragraph at the end of the Literature Review to clearly articulate the gap we bridge. We also added phrases like "within the studied hospital" to prevent overgeneralization.
